# Improved early and late continence following robot‐assisted radical prostatectomy with concurrent bladder neck fascial sling (RoboSling)

**DOI:** 10.1002/bco2.225

**Published:** 2023-02-15

**Authors:** Scott Leslie, Stuart Jackson, Mark Broe, Danielle C. van Diepen, Christina Stanislaus, Daniel Steffens, George McClintock, Sia Kim, Nicola Jeffery, Jeremy Fallot, Nariman Ahmadi, Arthur Vasilaras, Paul Sved, Lewis Chan, Ruban Thanigasalam

**Affiliations:** ^1^ RPA Institute of Academic Surgery (IAS) Royal Prince Alfred Hospital Camperdown New South Wales Australia; ^2^ Department of Urology Royal Prince Alfred Hospital (RPAH) Camperdown New South Wales Australia; ^3^ The University of Sydney Camperdown New South Wales Australia; ^4^ Chris O'Brien Lifehouse (COBL) Camperdown New South Wales Australia; ^5^ Department of Urology Concord Repatriation General Hospital Concord New South Wales Australia; ^6^ Surgical Outcomes Research Centre (SOuRCe) Camperdown New South Wales Australia

**Keywords:** incontinence, intraoperative sling, prostate cancer, prostatectomy, robotic surgery

## Abstract

**Objective:**

To describe a novel RoboSling technique performed at the time of robot‐assisted radical prostatectomy (RARP) and its utility for enhancing urinary function recovery postoperatively.

**Materials and Methods:**

The surgical technique involves harvesting a vascularised, fascial flap from the peritoneum on the posterior aspect of the bladder. Following completion of prostatectomy, the autologous flap is tunnelled underneath the bladder and incorporated into the rectourethralis and vertical longitudinal detrusor fibres at the posterior bladder neck with a modified Rocco suture. After urethra‐vesical anastomosis is completed, the corners of the flap are hitched up to Cooper's ligament bilaterally with V‐Loc sutures, tensioned and secured creating a bladder neck sling. A prospective, longitudinal cohort study was performed of 193 consecutive patients undergoing RARP between December 2016 and September 2019. The first 163 patients underwent standard RARP, and the last 30 patients had the RoboSling technique performed concurrently. Continence outcomes were the primary outcomes assessed using pad number and Expanded Prostate Cancer Composite (EPIC)‐urinary domain questionnaire. Operative time (OT), estimated blood loss (EBL), complications and oncological outcomes were secondary outcomes.

**Results:**

The two groups were comparable for demographics and clinicopathological variables. At 3 months, zero pad usage (*p* = 0.005) and continence rates, defined as EPIC score ≥ 85 (*p* = 0.007), were both higher in the RoboSling group. EBL, complication rate and positive surgical margin rate did not differ between the two groups. Superior zero pad usage was observed at 1 year in the RoboSling group (*p* = 0.029). The RoboSling technique added on average 16 min to OT.

**Conclusions:**

The RoboSling procedure at the time of RARP was associated with earlier return to continence without negatively impacting other postoperative outcomes. This improvement in continence outcomes was maintained long term.

## 1 INTRODUCTION

Radical prostatectomy offers excellent localised prostate cancer control and long‐term survival rates. Despite this success, the morbidity of prostatectomy has meant that urinary incontinence can be a major issue of prostate cancer survivorship.^1^, ^2^

Recovery of continence at 12 months following radical prostatectomy ranges from 60% to 93%, depending on the criteria used.^2^, ^3^, ^4^ Early continence rates are substantially lower than this, varying between 28% and 74%,^4^, ^5^ and can have a deleterious effect on quality of life (QoL) for these patients.^6^

Robotic‐assisted radical prostatectomy (RARP) demonstrates similar oncologic outcomes as open radical prostatectomy (ORP) or laparoscopic radical prostatectomy (LRP).^7^, ^8^ There is evidence of improved 12‐month urinary continence rates for RARP compared with ORP or LRP, although the overall quality of studies was poor.^9^ Furthermore, there was no consensus on the definition of continence with some studies using *zero* pad usage to define continence and others allowing the use of a *safety pad* for patients still considered continent.^9^ The only Level 1 evidence comparing functional outcomes between RARP and ORP did not demonstrate any difference in urinary continence either in the short‐term or in 24 months between the two groups.^10^ A recent randomised controlled trial (RCT) by Stolzenburg et al. demonstrated superior early continence rates following RARP compared to LRP, with continence rates at 3 months, 54% and 36%, respectively.^11^

A number of novel reconstructive and prophylactic surgical techniques have been described in the literature to improve continence.^12^, ^13^ Concurrent intraoperative placement of slings during RARP is one such concept.^14^, ^15^, ^16^, ^17^ Despite promising cohort data and a previous ORP trial demonstrating prophylactic sling benefit, recent randomised RARP trials have demonstrated no early continence benefit for non‐vascularised slings.^16^, ^17^

To our knowledge, there are no surgical techniques yet demonstrated during RARP that have used a *vascularised*, autologous sling, which supports the bladder neck and vesicourethral anastomosis. The objective of this study was to detail our technique and to assess its efficacy on enhancing urinary continence after robotic prostatectomy.

## 2 ROBOSLING TECHNIQUE

**FIGURE 1:**
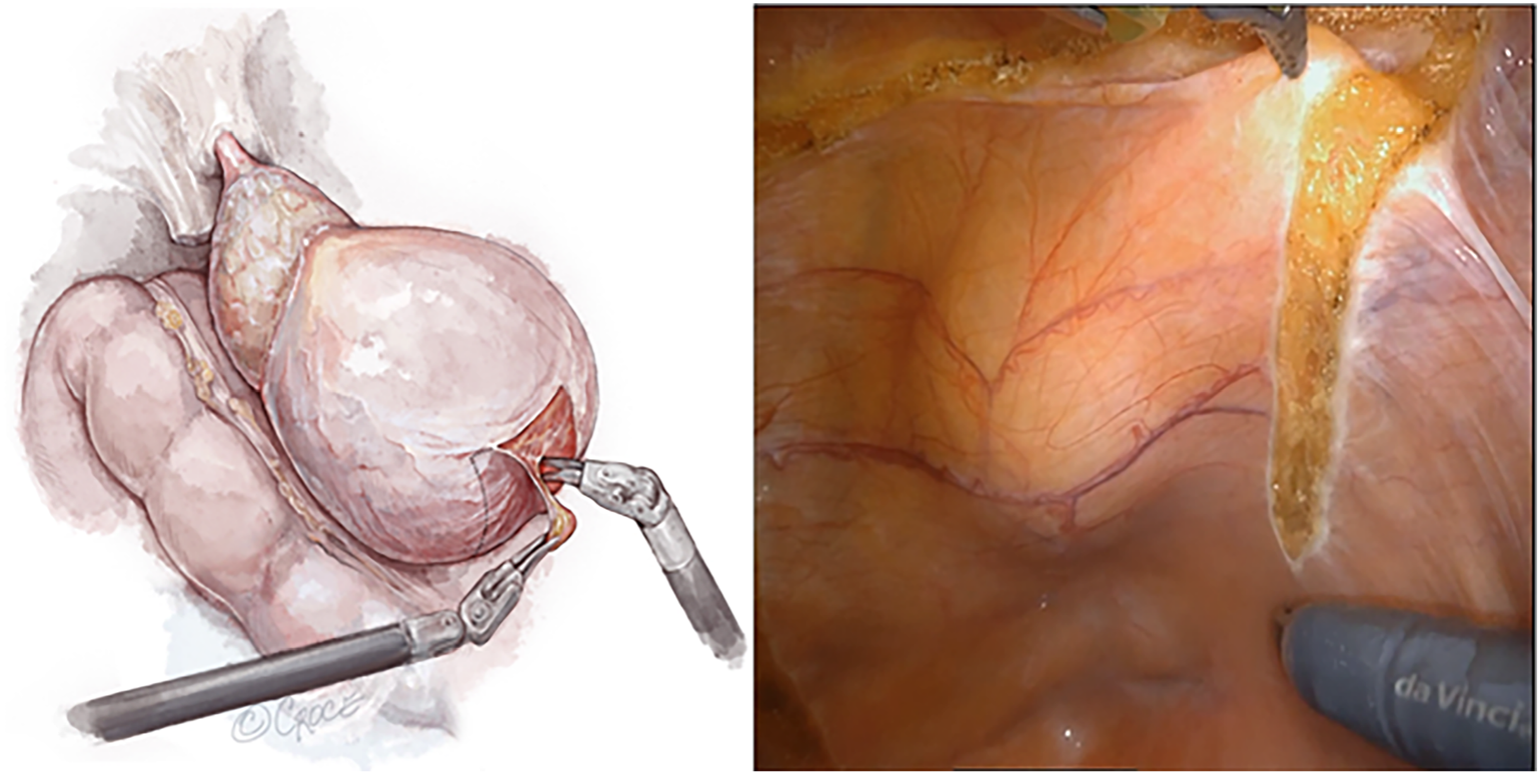
Harvesting of sling from the posterior bladder peritoneum

The RoboSling technique utilises an autologous vascularised fascial flap of the peritoneum placed underneath the urethrovesical anastomosis and suspended to the inguinal ligament of Cooper (pectineal ligament) with V‐Loc barbed sutures (Medtronic®, Minneapolis, USA), creating a supportive sling incorporated into the rectourethralis and bladder neck. RARP is performed via a transperitoneal approach with a standard 4 robotic port positioning. After robot docking, a rectangular‐shaped flap of the peritoneum on the posterior aspect of the bladder is dissected off the detrusor muscle (Figure 1). The flap is mobilised with a broad base to maintain its vascularity, vas deferens and seminal vesicles exposed bilaterally, and the posterior plane between Denonvilliers' fascia and the prostate is dissected towards the apex.

**FIGURE 2:**
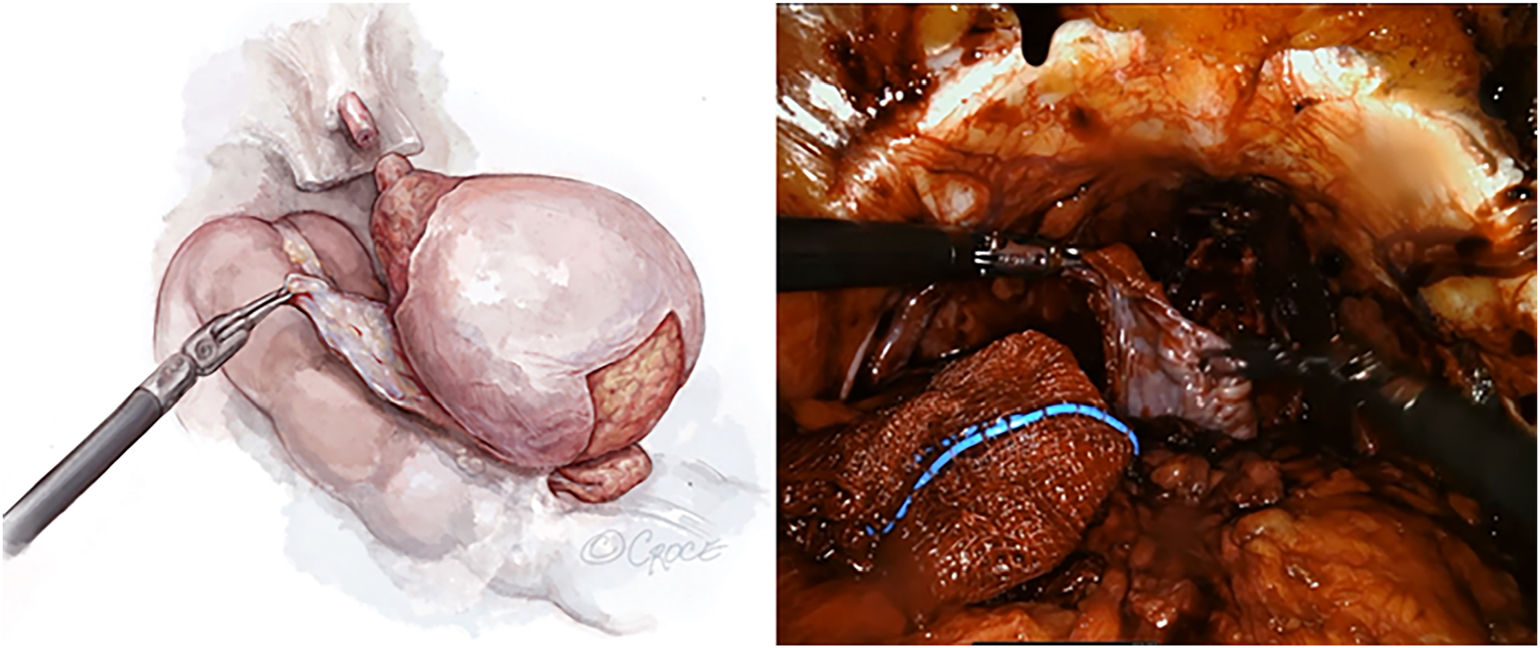
Tunnelling underneath the bladder post prostatectomy

**FIGURE 3:**
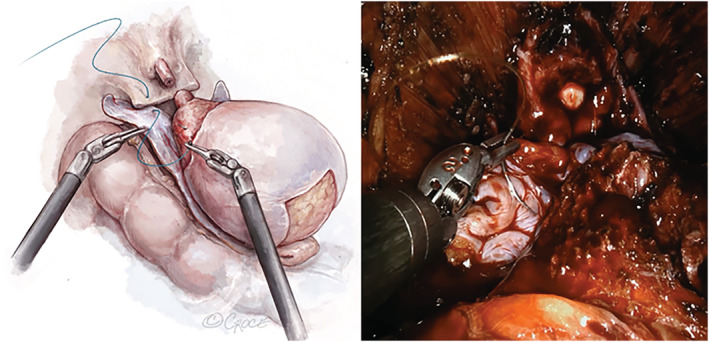
Incorporation of the sling into the Rocco suture

After the prostate has been removed, any lymph node dissection performed and haemostasis achieved, the peritoneal flap is transferred underneath the bladder (Figure 2). It is incorporated into the rectourethralis and bladder neck as a ‘modified Rocco Stitch’ using a 3‐0 V‐Loc suture (Figure 3).

**FIGURE 4:**
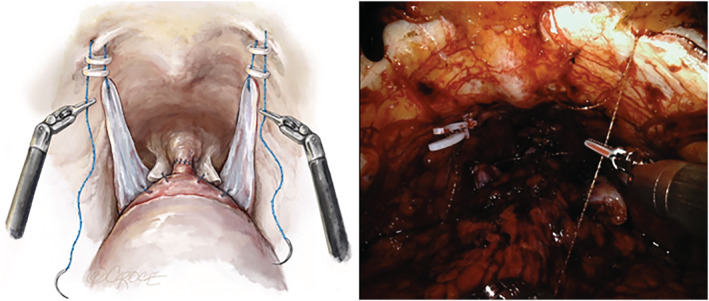
Tensioning flap laterally to pectineal ligament

The vesicourethral anastomosis is completed in usual fashion. Sequentially, at each corner of the flap, a 3‐0 V‐Loc suture is passed and hitched to the inguinal ligament of Cooper on each side (Supporting Information Figure S1)—these are tensioned by pulling on the suture and securing with two Hem‐o‐lok clips (Teleflex®, Philadelphia, USA) (Figure 4). As the V‐Loc sutures are tensioned, the bladder neck, rectourethralis and urethrovesical anastomosis are lifted upwards and supported by the RoboSling. Tensioning is performed with an 18Fr Foley catheter in place, so as not to over tension the sling.

## 3 MATERIALS AND METHODS

### 3.1 Study patient selection

A prospective, non‐randomised cohort study was undertaken to assess technique utility and safety. Demographic, oncological and functional outcomes have been prospectively collected. The outcomes of the first 193 men who underwent RARP between December 2016 and September 2019 were analysed—163 standard RARP compared to 30 patients (intervention group) who also had the novel RoboSling technique performed. Royal Prince Alfred Hospital (RPAH) is a tertiary referral centre and public teaching hospital with trainees performing supervised steps of procedures. All basic prostatectomy steps were identical in both groups except for inclusion of RoboSling. Operative decisions on bladder neck preservation, nerve‐sparing and lymph node dissection were not influenced by the RoboSling technique. All patients who participated provided a written informed consent under ethical approval of the Sydney Local Health District Human Research Ethics Committee (LNR/18/RPAH/266‐X18‐0195).

### 3.2 Primary outcomes

Primary outcomes of pad usage were evaluated at 3 and 12 months postoperatively. Pad usage at each time point was measured as average pad use per day for the preceding week. Patients filled in the Expanded Prostate Cancer Composite (EPIC)‐urinary domain questionnaire^18^ at 3 and 12 months postoperatively. This is a validated self‐assessment questionnaire composed of 12 questions that address continence, as well as other voiding problems. The EPIC score ranges from 0 to 100 points with higher scores signifying better quality urination; however, it lacks a clear definition differentiating continence from incontinence. Previous studies have indicated that an EPIC score ≥85 is associated with men being satisfied with their urinary continence, and therefore, in our study, a cut‐off score of 85 is used to compare outcomes.^19^

### 3.3 Secondary outcomes

Secondary outcomes included QoL, operative time (OT), positive surgical margins (PSM), length of stay (LOS), estimated blood loss (EBL) and complication rate. Short Form‐36 (SF‐36) questionnaire is utilised to assess QoL and is completed at 6 weeks and 12 months.

### 3.4 Statistical analysis

Baseline variables were compared between groups. Categorical variables were compared using the chi‐squared test (frequencies and proportions). Continuous variables were compared using the *t*‐test (means) or Wilcoxon 2‐sample test (median). All statistical analyses were performed using SPSS version 25 (SPSS Inc., Chicago, IL, USA).

## 4 RESULTS

The study consisted of 30 patients who underwent RARP with concurrent RoboSling and 163 without the RoboSling. Baseline characteristics did not differ between the two groups (Table 1). A total of 151 patients (78% response rate) filled in their 3‐month postoperative questionnaire, and 141 patients (73% response rate) filled in their 12‐month questionnaire. The continence outcomes for the study population are outlined in Table 2. At 3 months, zero pad usage in the RoboSling group was significantly higher (44% vs. 16.5%, *p* = 0.005). The RoboSling group's EPIC score was higher at 3 months (mean 62 vs. 43, *p* = 0.008). The percentage of EPIC score ≥85 at 3 months was higher with RoboSling placement (28% vs. 7%, *p* = 0.007), which is further evidence of better early continence.

**TABLE 1:** Baseline demographic data

Variable	RoboSling (*n* = 30)	No RoboSling (*n* = 163)	*p* value
Age (years)			
Mean (SD)	62.9 (9.2)	65.2 (6.9)	0.221
BMI (kg/m^2^)			
Mean (SD)	26.9 (5.9)	27.2 (4.6)	0.401
Preoperative ASA Score			
1	2 (9%)	25 (16%)	0.461
2	16 (70%)	99 (64%)	
3	4 (17%)	30 (19%)	
4	1 (4%)	1 (1%)	
Preoperative PSA level (ng/ml)			
Median (IQR)	7 (4.5–8.7)	6.8 (5.0–9.6)	0.168
Preoperative Gleason score			
≤ 6	4 (13%)	10 (6%)	0.134
7	20 (67%)	117 (72%)	
≥ 8	6 (20%)	36 (22%)	
Prostate volume (ml)			
Mean (SD)	38.1 (18.9)	38.2 (20.0)	0.314

Abbreviations: ASA, American Society of Anesthesiologists; IQR, interquartile range; PSA, prostate‐specific antigen.

**TABLE 2:** Continence outcomes

Variable	3 months			12 months		
	RoboSling (*n* = 18)	No RoboSling (*n* = 133)	*p* value	RoboSling (*n* = 18)	No RoboSling (*n* = 123)	*p* value
Zero pad (%)	8 (44.4)	22 (16.5)	0.005	13 (72.2)	55 (44.7)	0.029
≥ 1 Pad (%)	10 (55.6)	111 (83.5)		5 (27.8)	68 (55.3)	
EPIC score mean (SD)	62 (33)	43 (27)	0.008	73 (15)	65 (27)	0.237
EPIC ≥85 (%)	5 (28)	9 (7)	0.007	8 (47)	38 (39)	0.520
EPIC <85 (%)	13 (72)	113 (93)		9 (53)	60 (61)	

Abbreviation: EPIC, Expanded Prostate Cancer Composite.

**FIGURE 5:**
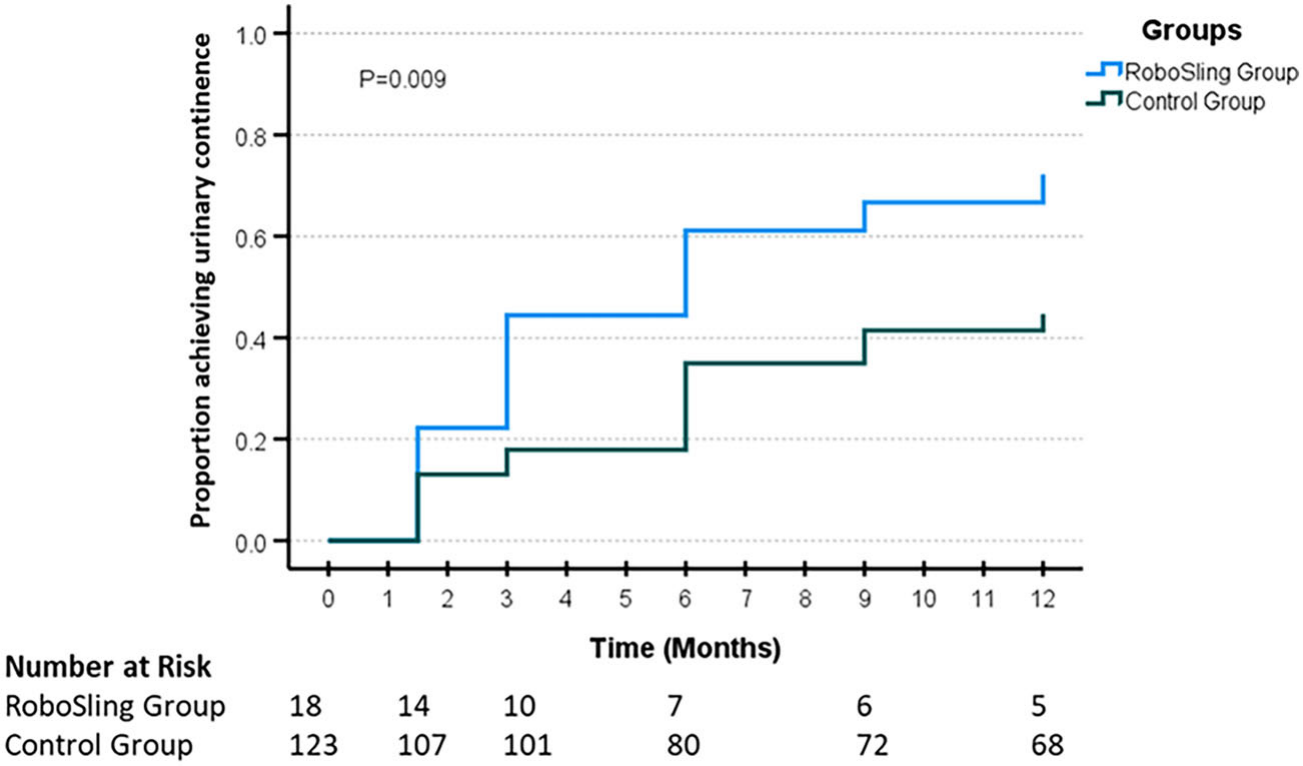
Continence indicated by zero pad usage over time

With longer follow‐up, the continence outcomes of the two groups improved as expected (Figure 5). At 12 months, the mean EPIC scores were not significantly different between the two groups (73 vs. 65, *p* = 0.237). The number of patients with EPIC ≥85 was also similar between the two groups at 1 year. Despite no real difference in EPIC score, men wearing zero pads at 12 months in the RoboSling group were significantly higher (72.2% vs. 44.7%, *p* = 0.029).

There were no significant differences demonstrated for EBL, LOS or pathological outcomes, including no difference in the PSM rate (Table 3). As expected, performing the RoboSling procedure added to the surgical time due to the extra steps required. The mean OT was 16 min longer in the RoboSling cohort (*p* = 0.047), and the mean console time was 14 min longer (*p* = 0.02). The complication rate was not significantly different between the RoboSling group and the control group (Supporting Information Table S1). The Grade IIIb Clavien‐Dindo complication in the RoboSling group was surgical reduction and closure of small bowel herniation through the umbilical port site at 7 days postoperatively. The patient subsequently made an uneventful recovery following this procedure. None of the patients that underwent the RoboSling procedure experienced urinary retention following catheter removal.

**TABLE 3:** Perioperative and pathological outcomes

Variable	RoboSling (*n* = 30)	No RoboSling (*n* = 163)	*p* value
Prostatectomy specimen Gleason score			
≤6	4 (13%)	6 (4%)	0.069
7	20 (67%)	134 (82%)	
≥8	5 (17%)	23 (14%)	
Not assessable	1 (3%)	0 (0%)	
Extra prostatic extension			
pT3 (%)	10 (33.3%)	80 (49.1%)	0.163
Positive surgical margins			
*n* (%)	6 (20.0%)	49 (30.1%)	0.379
Complication rate			
*n* (%)	2 (6.7%)	6 (4.1%)	0.361
Estimated blood loss (mls)			
Mean (SD)	232 (184)	273 (159)	0.811
Total operative time (h:min)			
Mean (SD)	4:05 (0:41)	3:49 (0:36)	0.047
Console time (h:min)			
Mean (SD)	3:08 (0:40)	2:54 (0:30)	0.020
Length of stay (days)			
Mean (SD)	2.1 (1.7)	2.4 (2.3)	0.347

Although differences are seen between the RoboSling group and the control group with regard to continence outcomes, there was no difference in overall QoL. The SF‐36 scores, for both the physical component score (PCS) and the mental component score (MCS), at 6 weeks and 12 months were equivalent between the RoboSling group and the control group (Supporting Information Table S2).

## 5 DISCUSSION

This prospective study assesses the effect of the RoboSling—a novel, autologous vascularised fascial sling placed during RARP—and its effects on postoperative urinary incontinence. We report a beneficial effect with RoboSling placement on pad usage and continence rates at 3 months and 12 months. Significantly, there was no associated increase in complications or PSM rate.

The rationale for the use of a vascularised autologous sling positioned during RARP is to assist the restoration of vesicourethral and pelvic anatomical support, with prevention of bladder descent, and improvement of outlet resistance via a reduced posterior urethrovesical angle and increased exposure to intra‐abdominal pressure. This hypothesis could be supported by our initial results.

Jorion et al. first described concurrent autologous sling placement during radical retropubic prostatectomy in 1997.^3^ Utilising a rectus fascial sling, continence rates were significantly higher for the sling cohort at 2 months, compared with non‐sling control (93% vs. 70%; *p* = 0.04). A similar retrospective study using rectus fascia found no difference in pad usage at 6 and 12 months, though with a statistically significant increase in urethral stricture (35% vs. 14%; *p* = 0.001) for the sling group.^4^ However, a significantly higher proportion of men in the sling group had previous radiation therapy (0.017). More recently, a retrospective RARP study using autologous slings made of median umbilical ligament/vas deferens demonstrated no effect on time to one pad (*p* = 0.24) or no pads (*p* = 0.20).^5^ Population characteristics of the intervention group may explain the higher risk of postoperative incontinence due to the impact of non‐nerve‐sparing, larger bladder necks and shorter urethral lengths—factors associated with worse incontinence.^6^ A RARP sling technique using the retrotrigonal muscular layer demonstrated a significant improvement in early continence rates at 1 (*p* = 0.0049) and 4 weeks (*p* = 0.035).^20^ Jones et al. examined the use of a non‐autologous sling during ORP with the use of either the porcine small intestine submucosa or polyglactin mesh underneath the anastomosis.^21^ Continence rates were higher in the sling cohort at 12 weeks (93% vs. 47%), although this was a small retrospective study.

Some RCTs have demonstrated benefits in urinary function with the use of autologous intraoperative slings. The use of an anterior rectus fascial sling during ORP had lower incontinence rate than control (17.5% vs. 43.5%, *p* = 0.010) with a statistically shorter time to achieve continence.^22^ The first RCT of RARP with a concurrently placed autologous sling using the vas deferens had less pads used per day (0.4 vs. 1.1; *p* = 0.01) and higher early zero pad rate at 30 days (76% vs. 46%; *p* = 0.03) but also maintained at 3, 6 and 12 months.^15^ Contrary to these positive results, Nguyen et al.'s randomised RARP study using the vas deferens did not demonstrate any benefit in postoperative continence, early or late.^17^

RCTs utilising non‐autologous slings have produced mixed results. The use of a single suspended VICRYL suture after double‐layered posterior rhabdosphincter reconstruction demonstrated a subjective benefit for patients as well as objective data of urinary incontinence 4 weeks after RARP in the sling group.^14^ Bahler et al. used the porcine small intestinal submucosa secured to the pubic bone but failed to demonstrate any statistically significant improvement in urinary continence.^16^ Overall, there is low to moderate certainty evidence that concurrent sling placement during prostatectomy is generally favourable for short‐term improvement but make no difference in long‐term continence outcomes.^13^

Our study describes a novel RoboSling technique that involves the first reported use of an autologous fascial flap of the peritoneum, utilised as a prophylactic sling during RARP. Compared to the previous published techniques, the RoboSling is unique as the sling material is *vascularised* and supports the bladder neck, urethrovesical anastomosis and pelvic floor over a broad surface area by incorporating the sling with a ‘modified Rocco’ stitch. The RoboSling procedure only adds slightly to the surgical time, owing to the additional steps necessary to create the sling. However, with this technique, zero pad use was significantly improved in the RoboSling group both at 3‐months follow‐up and at 12‐months follow‐up, showing the early benefit, which also extended to longer follow‐up at 1 year after surgery. Improved early incontinence was also reinforced with better EPIC scores at 3 months. The RoboSling was not difficult for the trainees to perform and added on average 14 min to console time, which is felt to be justifiable. Furthermore, there is a learning curve for surgeons and trainees utilising this novel technique, and it is anticipated that OT would improve as experience with the RoboSling increased. It did not result in any increased rate of complications in the sling cohort. Importantly, there were no cases of ureteric injury, bladder injury or urinary retention. Urinary tract infection occurred once in each group.

There are several limitations that should be noted for this study. Confounders such as prostate volume to pelvic volume ratio, preoperative urethral length and angle and bladder neck size may affect continence outcomes and were not assessed in this cohort of patients. Second, while questionnaires were provided prospectively, patients were completing these with retrospective recall of the preceding period, with potential for associated bias. Third, in our study, the rates of continence, particularly in the control group, are lower than other rates published in the literature^18^; however, it should be noted that a strict definition of ‘*zero* pads’ was used to define continence, and furthermore, as a public teaching hospital, these cases were predominantly performed with the trainee or fellow as the primary surgeon on the console. Additionally, there continues to be no clear consensus on a measurable definition of continence, and the use of ‘pad number’ as a measure of the degree of incontinence may not be reliable. For example, an incontinent patient who loses urine but refuses to wear pads could hardly be considered continent. Conversely, a careful man who hardly leaks but changes his pads multiple times daily to maintain hygiene may be misinterpreted to be reliant on pads when he is actually not. Therefore, a more accurate measure of incontinence is 24‐hour pad weight as it better quantifies the amount of leakage. 24‐hour pad weight in combination with QoL measures (EPIC, SF‐36) would be the optimal tools for any future studies to quantify urinary incontinence outcomes following prostate surgery.^23^ Finally, as this was a non‐blinded study, there is potential for bias as patients' knowledge they are receiving a RoboSling could influence their symptom scores and contribute to them wearing less pads. These limitations are addressed in the blinded, RCT that is currently underway at our institution.

## 6 CONCLUSION

In summary, the RoboSling technique is a novel, easily performed intraoperative sling at the time of RARP that demonstrates improved rates of continence both in the short term and in 1‐year following surgery without a higher complication or positive surgical margin rate. A randomised controlled study is now enrolling at our institution to further assess the benefits of this technique.

## CONFLICT OF INTEREST

None of the authors or institutions have any disclosures to declare in relation to this study.

## AUTHOR CONTRIBUTIONS

All authors contributed to the production of this manuscript. The contributions of the authors are included below:

Conception and design: Scott Leslie, Daniel Steffens, Nicola Jeffery, Ruban Thanigasalam, Nariman Ahmadi and Mark Broe. Acquisition of data: Scott Leslie, Stuart Jackson, Jeremy Fallot, Danielle C van Diepen, Christina Stanislaus, Arthur Vasilaras, Paul Sved and Ruban Thanigasalam. Analysis and interpretation of data: Scott Leslie, Jeremy Fallot, George McClintock and Ruban Thanigasalam. Drafting of the manuscript: Scott Leslie, Stuart Jackson, Jeremy Fallot, George McClintock, Sia Kim and Mark Broe. Critical revision of the manuscript for important intellectual content: Daniel Steffens, George McClintock, Nicola Jeffery, Ruban Thanigasalam, Nariman Ahmadi and Mark Broe. Statistical analysis: Jeremy Fallot, Danielle C van Diepen and Daniel Steffens. Administrative, technical, or material support: Christina Stanislaus, Daniel Steffens and George McClintock. Supervision: Lewis Chan.

## Supporting information


**Figure S1.** The corners of the sling are sutured laterally.
**Table S1.** Postoperative complications.
**Table S2.** Quality of life outcomes (SF‐36)
