# Supplementary material for: Improved early and late continence following robot‐assisted radical prostatectomy with concurrent bladder neck fascial sling (RoboSling)
Source: BJUI Compass. 2023 Feb 15;4(5):597–604. doi: 10.1002/bco2.225 (PMC10447214; doi:10.1002/bco2.225)
Supplement: Supplementary file 1 — Figure S1. The corners of the sling are sutured laterally. Table S1. Postoperative complications. Table S2. Quality of life outcomes (SF‐36) [file BCO2-4-597-s001.docx]

| **Variable** | **RoboSling (n=30)** | **No RoboSling (n=163)** | **P-value** |
| --- | --- | --- | --- |
| **Age (years)** |  |  |  |
| Mean (SD) | 62.9 (9.2) | 65.2 (6.9) | 0.221 |
| **BMI (kg/m^2^)** |  |  |  |
| Mean (SD) | 26.9 (5.9) | 27.2 (4.6) | 0.401 |

|  | **Clavien-Dindo Grade** | **Description** |
| --- | --- | --- |
| **RoboSling (n=2)** | II | UTI |
|  | III b | Wound dehiscence |
| **No RoboSling (n=6)** | I | Ileus |
|  | II | HDU Admission for tachycardia. Normal Hb |
|  | II | UTI |
|  | II | Lymphocele |
|  | IV a | ICU Admission for unknown hypotension. |
|  | IV a | Respiratory failure |

**Supplementary Table A**. Postoperative complications

|  |  | **Mean (SD)** | **p-value** |
| --- | --- | --- | --- |
| **6 Weeks PCS** | **RoboSling (n=25)** | 46.6 (13.1) | 0.316 |
|  | **Control (n=134)** | 43.1 (16.1) |  |
| **1 Year PCS** | **RoboSling (n=14)** | 51.4 (10.9) | 0.766 |
|  | **Control (n=93)** | 52.1 (6.8) |  |
| **6 Weeks MCS** | **RoboSling (n=25)** | 46.9 (13.7) | 0.085 |
|  | **Control (n=134)** | 40.7 (17.1) |  |
| **1 Year MCS** | **RoboSling (n=14)** | 50.8 (6.2) | 0.457 |
|  | **Control (n=93)** | 48.8 (9.9) |  |

PCS: Physical Component Score; MCS: Mental Component Score

**Supplementary Table B.** Quality of life outcomes (SF-36)
